# Effect of the Great Recession on regional mortality trends in Europe

**DOI:** 10.1038/s41467-019-08539-w

**Published:** 2019-02-08

**Authors:** Joan Ballester, Jean-Marie Robine, François R. Herrmann, Xavier Rodó

**Affiliations:** 1 Climate and Health Program, Barcelona Institute for Global Health (ISGlobal), Barcelona, Catalonia Spain; 2 Institut National de la Santé et de la Recherche Médicale (INSERM), Montpellier, France; 3 École Pratique des Hautes Études, Paris, France; 4 Division of Geriatrics, Department of Rehabilitation and Geriatrics, Geneva University Hospitals and University of Geneva, Thonex, Switzerland; 5 ICREA, Barcelona, Catalonia Spain

## Abstract

Previous studies have consistently shown the recurrent relationship between macroeconomic cycles and changes in mortality trends, so that recessions are generally associated with periods of faster life expectancy rise, and periods of economic growth with slower reductions or even increases in mortality trends. Here we analyze the link between annual per capita estimates of gross domestic product and daily atmospheric temperatures and standardized death rates for a large ensemble of European regions to describe the effect of the Great Recession on annual and seasonal changes in all-cause human mortality trends. Results show that the countries and regions with the largest (smallest) economic slowdown were also those with the largest (smallest) strengthening of the declining mortality trend. This procyclical evolution of mortality rates is found to be stronger during the cold part of the year, showing that it also depends on the seasonal timing of the underlying causes of death.

## Abstract (web summary)

Country difference in macroeconomic cycles-mortality changes relationships has been rarely explored. Here the authors studied the relationship between 2008 recession and daily mortality counts for EU countries and revealed a significant relationship between macroeconomic cycles and mortality trends.

## Introduction

On the long run, there is a well-established positive association between economic growth and life expectancy^1^. Although the direction of causation between wealth and longevity is still debated, people in developed economies live clearly longer than people in less advanced countries^2^. Thus, any eventual negative impact of economic growth is counterbalanced by a set of long-term beneficial effects, such as those derived from investments in education, social services, research, and public health systems. Apart from exceptional cases^3^, developed societies are generally characterized by a path of economic growth and longevity rise (e.g., ref. ^4^).

Although the sustained decline in human mortality is continuously increasing the life expectancy^5,6^, on the short run, several authors since ref. ^7^ have investigated if mortality rates are procyclical (i.e., greater declines in mortality are observed during economic slowdowns) or countercyclical (i.e., during expansions). The question is still hotly debated and subject to vivid controversy, e.g., refs. ^8–15^.

On the one hand, economic crises are associated with job displacements and increasing unemployment rates, which generally worsen the life conditions of the most vulnerable groups^16–18^. In addition, recessions normally evolve into crises of national public debt, such as in Europe after 2008, which force governments to apply austerity programs^19^, leading in some cases to reductions in the use of medical care and routine medical checkups^20,21^. It is generally believed that these and other similar factors have a dominant impact on human mortality trends. Some authors have claimed that expansions are associated with delayed (up to a few years) beneficial effects for vulnerable people through an increase in governmental expenditure and the reinforcement of health care systems^22,23^, and more recently, a few studies described several countercyclical indicators^24^.

On the other hand, some studies have criticized the methodologies used therein^25–28^, or shown evidence in the opposite direction^4,11,29–31^, pointing to the procyclical evolution of mortality risks^20^. Although most of the available evidence has explored this result in developed countries^4,17,29,32,33^, there is also a growing body of literature confirming results in developing societies^26,34,35^. The relationship has also been shown for different periods and economic cycles^31,36^, although it is not clear yet if the strength of the procyclical behavior has decreased in recent times ^9,37–39^.

Several explanations have been proposed to explain the procyclical evolution of mortality, such as a reduction in environmental pollution and driving and occupational deaths due to decreased economic activity and employment^11^. For example, a one percentage point increase in unemployment is associated with a reduction in traffic mortality of 3% in the United States^20^, or 2.1% in the OECD countries^32^. Moreover, ref. ^40^, showed that the relationship between mortality and unemployment was attenuated by 17% after controlling for several major air pollutants. Other studies have emphasized that several lifestyle variables that influence health are also procyclical; for instance alcohol consumption, smoking, obesity, and physical inactivity usually decrease during recessions^41^. These mechanisms are not completely understood and subject to further investigation, but these relations seem to be associated with factors such as job-related stress^42^ and the fact that healthy habits are time-consuming^17^, and therefore less likely to be observed during periods of low unemployment and longer working days^43^.

Although these general associations are found to be consistent among economic cycles, fewer and less conclusive studies are available for other factors such as some causes of death, socioeconomic status, sex, or race^11,27^. For example, little evidence is available for mortality due to specific causes or affecting specific groups of the population, and countercyclical effects are often found for suicides^26,44,45^. In addition, due to the scarcity and fragmentation of daily time series of mortality data, no study has ever analyzed the seasonality of mortality to show whether it is found to be procyclical and/or countercyclical during the different phases of the calendar year.

Here we use Gross Domestic Product (GDP) estimations, daily temperatures, and mortality rates to describe the effect of the recent 2008 recession in Europe on annual and seasonal changes in all-cause human mortality trends. This analysis is done through the investigation of the associations between changes in mortality, macroeconomic conditions, and temperatures in a number of European regions, therefore indicating which are the countries and regions in which the economic slowdown associated with the 2008 recession had the largest effect on annual and seasonal mortality trends. Results show that the countries and regions with the largest (smallest) economic slowdown were also those with the largest (smallest) strengthening of the declining mortality trend. This procyclical evolution of mortality rates is found to be stronger during the cold part of the year, showing that it also depends on the seasonal timing of the underlying causes of death.

## Results

### Evolution of GDP during the Great Recession

The present work is aimed at comparing the mortality trends before and after the onset of the Great Recession in Europe. There is not, however, a definition of onset that can be generally applied to all the countries and regions here analyzed. Both the European Union and the Eurozone were officially in recession (i.e., negative seasonally-controlled real GDP by at least two consecutive quarters) between the third quarter of year 2008 (Q3-2008) and the second quarter of 2009 (Q2-2009), but the exact timing varied from one country to another^46^. Among the countries here analyzed, the recession appeared earlier in Italy (Q3-2007) and later in Belgium, Croatia, the Netherlands, Slovenia, Spain (Q4-2008), and the Czech Republic (Q1-2009), while it did not even technically appear in Poland. The end of this initial period of recession between years 2007 and 2010 was also heterogeneous, ranging from Q2-2009 in Germany and Portugal to Q1-2010 in Spain and Q3-2010 in Croatia.

**Fig. 1:**
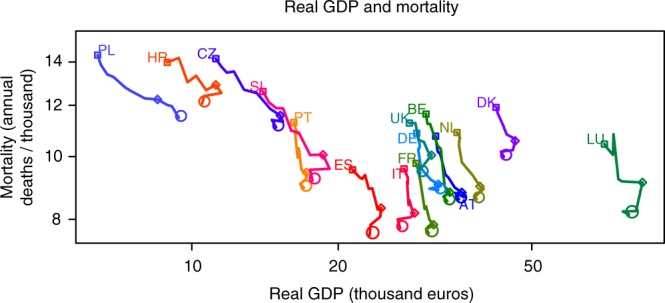
Recent evolution of macroeconomic and health indicators. The panel shows the evolution of real Gross Domestic Product (GDP, thousand euros) and age-standardized mortality (annual deaths per thousand). Squares and circles indicate the beginning and end of the study period, respectively. Diamonds correspond to year 2007. Country acronyms are: Austria (AT), Belgium (BE), Croatia (HR), the Czech Republic (CZ), Denmark (DK), France (FR), Germany (DE), Italy (IT), Luxembourg (LU), Netherlands (NL), Poland (PL), Portugal (PT), Slovenia (SI), Spain (ES), and the United Kingdom (UK)

Although there is obviously some degree of homogeneity between the recent macroeconomic evolution of the countries here analyzed, mainly due to the global impact of this particular crisis and the economic interdependence between the member states of the European Union, we cannot properly define periods of expansion and recession that are generally valid to all cases. In addition, the period in which countries were officially in recession before 2010 (i.e., the period for which we have daily mortality data) was relatively short and variable, which does not allow us to perform a comparative analysis between expansion and recession periods of comparable length. That is the reason why we computed correlations between changes in annual GDP and mortality trends. In this way, our results do not largely depend on the year chosen to characterize the transition from expansion to recession, but rather on the correlation between relative changes in GDP and relative changes in mortality trends, regardless of the sign of GDP changes occurred during the period chosen to represent the recession phase (e.g., the real GDP increased in Poland for all the years of the study period, see Fig. 1). Associations and correlations were separately computed for the ensemble of available countries and regions, hereafter referred to as spatial associations and correlations for simplicity. Please note that all correlations were weighted by the square root of the population^4^.

### Changes in GDP and mortality trends

Figure 1 and Tables 1 and 2 show the temporal evolution of real GDP and mortality by country. On the one hand, the real GDP increased between 2000 and 2010 in all the analyzed countries except Italy (−0.2%/year), with very large increases in Poland (+4.7%/year), the Czech Republic (+3.3%/year), Slovenia (+2.6%/year), and Croatia (+2.2%/year). Although the real GDP did not decrease between 2007 and 2010 (recession period) in Poland (+3.5% / year) and Germany (+0.0% / year), it increased between 2000 and 2007 (expansion period) in all the analyzed countries, and this increase was larger than the one in the recession period in all cases. As a result, all the countries experienced a relative slowdown of their economies. On the other hand, annual mortality decreased in both periods for all the countries. Nonetheless, the largest decrease is found during the recession period in nearly half of the countries (Denmark, Spain, Croatia, Luxembourg, Poland, and Slovenia), and during the expansion period in all the others (Austria, Belgium, the Czech Republic, Germany, France, Italy, the Netherlands, Portugal, and the United Kingdom).

**Table 1:** Real Gross Domestic Product (GDP, euros)

	Real GDP (euros)			Real GDP change (% per year)		
	Year 2000	Year 2007	Year 2010	2000 vs. 2007	2007 vs. 2010	2000 vs. 2010
Austria	31,700	35,900	35,400	1.9	−0.5	1.2
Belgium	30,300	33,900	33,500	1.7	−0.4	1.1
Czech Republic	11,200	15,200	14,900	5.1	−0.7	3.3
Germany	29,000	32,100	32,100	1.5	0.0	1.1
Denmark	42,200	46,200	43,800	1.4	−1.7	0.4
Spain	21,400	24,500	23,200	2.1	−1.8	0.8
France	28,900	31,400	30,700	1.2	−0.7	0.6
Croatia	8900	11,200	10,500	5.2	−2.1	2.2
Italy	27,300	28,700	26,800	0.7	−2.2	−0.2
Luxembourg	70,500	84,400	79,200	2.8	−2.1	1.2
Netherlands	35,100	39,100	38,500	1.6	−0.5	1.0
Poland	6400	8500	9400	4.7	3.5	4.7
Portugal	16,200	17,200	17,000	0.9	−0.4	0.5
Slovenia	14,000	18,600	17,700	4.7	−1.6	2.6
United Kingdom	28,000	31,100	29,500	2.2	−1.7	0.7

Average changes are shown as relative differences between indicated years (% per year)

**Table 2:** Age-standardized mortality (annual deaths per thousand)

	Mortality (annual deaths per thousand)			Mortality change (% per year)		
	Year 2000	Year 2007	Year 2010	2000 vs. 2007	2007 vs. 2010	2000 vs. 2010
Austria	10.756	8.804	8.588	−2.593	−0.815	−2.015
Belgium	11.636	8.811	8.531	−3.469	−1.061	−2.669
Czech Republic	14.168	11.576	11.097	−2.613	−1.380	−2.168
Germany	10.866	9.062	8.873	−2.372	−0.694	−1.834
Denmark	11.915	10.575	9.996	−1.606	−1.826	−1.611
Spain	9.543	8.327	7.560	−1.820	−3.070	−2.078
France	9.763	7.849	7.614	−2.800	−0.999	−2.201
Croatia	13.981	12.904	12.085	−1.540	−2.117	−1.695
Italy	9.576	8.183	7.755	−2.079	−1.742	−1.902
Luxembourg	10.458	9.123	8.141	−1.823	−3.589	−2.215
Netherlands	10.896	8.997	8.594	−2.490	−1.494	−2.113
Poland	14.348	12.255	11.480	−2.084	−2.107	−1.999
Portugal	11.288	9.453	8.952	−2.322	−1.765	−2.069
Slovenia	12.607	10.067	9.189	−2.878	−2.905	−2.711
United Kingdom	11.266	10.049	9.433	−2.159	−2.046	−2.034

Average changes are shown as relative differences between indicated years (% per year)

**Fig. 2:**
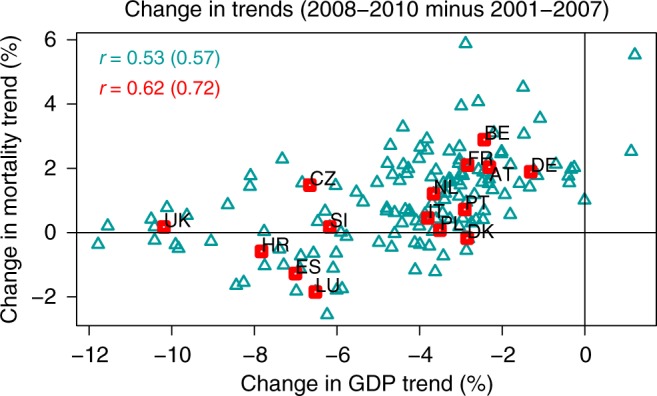
Recession minus expansion change in trends. The panel shows the difference (2008–2010 minus 2001–2007) in average relative year-to-year changes (%) of annual Gross Domestic Product (GDP) and age-standardized mortality by country (red squares) and region (cyan triangles). Correlations are depicted in the top-left corner (Pearson first, Spearman in brackets). Country acronyms are: Austria (AT), Belgium (BE), Croatia (HR), the Czech Republic (CZ), Denmark (DK), France (FR), Germany (DE), Italy (IT), Luxembourg (LU), Netherlands (NL), Poland (PL), Portugal (PT), Slovenia (SI), Spain (ES), and the United Kingdom (UK)

The relative year-to-year changes in annual GDP and mortality were averaged for the expansion and recession periods, and differences between periods (recession minus expansion) are depicted in Fig. 2 (see Methods). This figure highlights the procyclical behavior of mortality by showing that the countries and regions with the largest (smallest) economic slowdown were also those with the largest (smallest) strengthening of the declining mortality trend. The resulting Pearson correlation is equal to 0.62 and 0.53 when it is calculated for the ensemble of countries and regions, respectively. In the present work, we only provide Pearson correlations, although we must note that Spearman correlations are generally higher, i.e., 0.72 and 0.57, for the ensemble of countries and regions, respectively. The correlation method we use is found to be robust to the choice of the expansion and recession periods, given that as explained above, it is based on the correlation between relative changes in GDP and mortality trends. For example, the Pearson correlation is only slightly smaller when differences in GDP and mortality trends are calculated between 2001–2005 and 2006–2010, e.g., 0.62 and 0.46, for the ensemble of countries and regions, respectively (Supplementary Fig. 1).

### Procyclical behavior of mortality by temperature

**Fig. 3:**
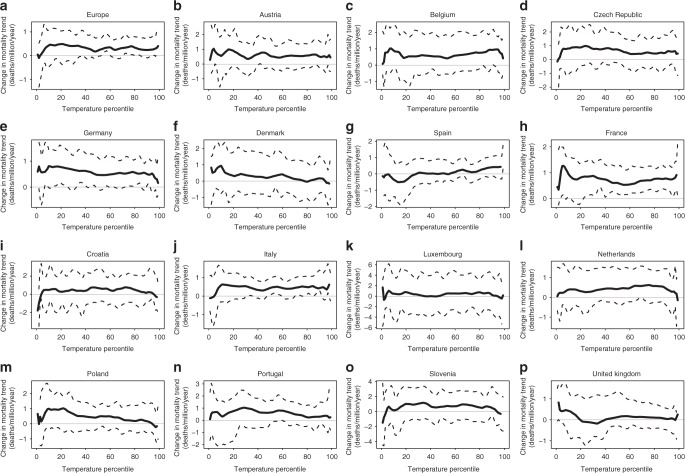
Change in mortality trends by temperature percentile. **a**–**p** show the change in the trend of age-standardized mortality by temperature percentile. For each location, percentiles of the daily temperature time series of the overall period 2000–2010 were computed and transformed into mortality estimations by using the daily temperature/mortality relationship from subperiods 2000–2003 (P1), 2004–2007 (P2), and 2008–2010 (P3). Differences in mortality between consecutive subperiods were used to infer expansion (P2 minus P1) and recession (P3 minus P2) trends (daily deaths per million per year). Solid lines correspond to the recession minus expansion difference in trends, and dashed curves to the 95% confidence interval

Our analysis went a step further by using daily temperatures and mortality rates to show the association between recession − expansion changes in annual GDP and mortality trends by temperature percentile, and in this way, to provide a description of the seasonal strength of the procyclical evolution of mortality shown in Fig. 2. We note that the mortality trends are in this case defined as mortality changes between consecutive subperiods, i.e., 2004–2007 minus 2000–2003 for the expansion, and 2008–2010 minus 2004–2007 for the recession (see Methods). Despite the noise associated with the relatively limited length of our daily records (i.e., 11 years of data for three subperiods), Supplementary Fig. 2 suggests that mortality differences between consecutive subperiods are to a first approximation independent from the value of temperature, and therefore, from any seasonal pattern. A closer look at the mortality trends of the expansion and recession periods, however, reveals some more subtle differences, for example between the warm and cold halves of the year (e.g., Germany, Denmark, and Spain; Fig. 3 e-g) or between the central and non-central temperature percentiles (e.g., Croatia, Slovenia, and the United Kingdom; Fig. 3 i,o,p). We note that this diversity is generally larger at the regional level (Supplementary Fig. 3).

**Fig. 4:**
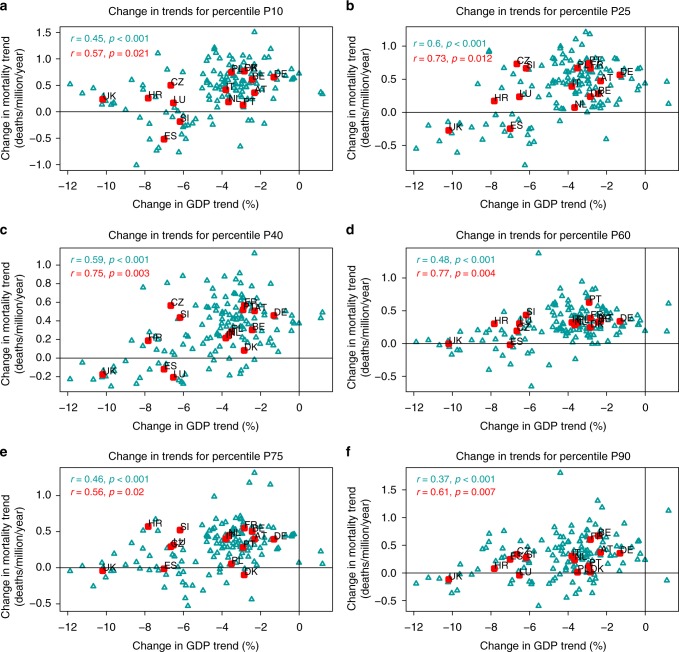
Change in trends by temperature percentile. Panels show the difference (recession minus expansion) in the trend of Gross Domestic Product (GDP, %) and age-standardized mortality (daily deaths per million per year) for temperature percentiles 10 (**a**), 25 (**b**), 40 (**c**), 60 (**d**), 75 (**e**), and 90 (**f**) by country (red squares) and region (cyan triangles). Pearson correlations and *p*-values are depicted in the top-left corner. Country acronyms are: Austria (AT), Belgium (BE), Croatia (HR), the Czech Republic (CZ), Denmark (DK), France (FR), Germany (DE), Italy (IT), Luxembourg (LU), Netherlands (NL), Poland (PL), Portugal (PT), Slovenia (SI), Spain (ES), and the United Kingdom (UK)

Figure 4 shows the analysis of the procyclical behavior of mortality for temperature percentiles 10, 25, 40, 60, 75, and 90, which are here shown as representative values for different phases of the calendar year. Pearson correlations and significance levels for the whole range of temperature percentiles are additionally provided in Supplementary Fig. 4. Despite the differences in the methodology used to estimate the change in mortality trends (see Methods), the average results in Fig. 4 and Supplementary Fig. 4 are qualitatively equal, and similar in magnitude, to those in Fig. 2 (cf. the solid curves with the horizontal dashed lines in Supplementary Fig. 4a). The match between the magnitude of the correlations highlights the fact that both methodologies are equivalent, and allows us to express the procyclical evolution of mortality as a function of the temperature percentile. Figure 4 and Supplementary Fig. 4 show that the strongest relationship between the change in economic growth and mortality trends is observed during the cold part of the year, between percentiles 10 and 70, while the magnitude of the association is generally smaller in summer, between percentiles 70 and 90. Importantly, mortality appears to be procyclical, and the association significant, for the whole range of temperature percentiles.

## Discussion

The procyclical evolution of mortality rates here shown involves both the strength and the length of the economic cycles, as well as the underlying base level of economic development, and arises from a complex interplay of a wide range of (sometimes opposing) effects of the periods of expansion and recession^47^. There are many mechanisms that have been claimed to explain this recurrent relationship between macroeconomic cycles and mortality oscillations. Periods of macroeconomic expansion are associated with increased pollution, occupational hazards, burnout and traffic injuries associated with increased industrial activities, travels, and consumption of various goods such as food, alcohol, and tobacco. Conversely, economic recession decreases air pollution and traffic deaths through reduced industrial activity, commuting, and alcohol consumption. Among all these factors, changes in atmospheric pollution^48^ and traffic and occupational fatalities^49^ are possibly the main drivers of the connection between changes in economic growth and mortality fluctuations^40^.

There is actually a body of evidence that shows that these rather immediate impacts are dominant on the short run, and largely counterbalance any eventual delayed effect in the opposite direction. Our results seem to fully support, both qualitatively and quantitatively, the association between macroeconomic slowdowns and greater declines in mortality. We must however note that our study is based on simultaneous relationships for the first years of the recession, when some European countries did not even start to implement major austerity measures. Nonetheless, our methodology based on relationships between changes in annual GDP and mortality trends, does not depend on the phase of the macroeconomic expansion-recession cycle, and therefore it already takes into account any eventual beneficial effect of increases in governmental expenditure during periods of economic growth.

Our results show that the countries and regions with the largest (smallest) economic slowdown were also those with the largest (smallest) strengthening of the declining mortality trend. We must emphasize that this result is only generally valid as a relative comparison between societies. When we analyze countries individually, the largest annual mortality decrease is found during the recession period in nearly half of the cases (Denmark, Spain, Croatia, Luxembourg, Poland, and Slovenia), and during the expansion period in all the others (Austria, Belgium, the Czech Republic, Germany, France, Italy, the Netherlands, Portugal, and the United Kingdom; Tables 1 and 2). Note that a similar classification of countries has been recently described in ref. ^4^ for life expectancy at birth and unemployment rate between periods 2004–2007 (expansion) and 2007–2010 (recession, see Table 1 therein). This is also consistent with single country analyses such as the one in ref. ^11^, which showed that all-cause mortality in Spain (i.e., a country of the first group above) decreased at a faster pace after the onset of the economic crisis (2008–2011) than before (2004–2007).

We further explored the association between pre- vs. post-crisis changes in GDP and mortality trends by using daily temperatures and mortality rates (Supplementary Fig. 2) to express this association as a function of the temperature percentile, and in this way, to provide a description of the seasonal dependence of the association, i.e., results corresponding to cold (warm) percentiles show the association between trend changes for the coldest (warmest) days and months of the calendar year (Fig. 4 and Supplementary Fig. 4). We must clarify that we did not aim to analyze here the fraction of deaths that are directly attributed to cold or warm temperatures, i.e., temperature-related mortality, which is typically modeled through Distributed Lag Nonlinear Models (DLNM^50,51^). Instead, we wanted to refine the analysis by showing the seasonal strength of the association between changes in GDP and mortality trends, and this is here done by means of the temperature percentiles. As a result, we did not explicitly filter out or control for the main confounders of temperature-related mortality, i.e., the seasonality and the long-term trend, precisely because these are the two main factors that we here seek to describe. Nonetheless, no systematic seasonal pattern was found in the mortality trends (Fig. 3 and Supplementary Fig. 3), given that they are essentially cancelled out when consecutive subperiods are subtracted (Supplementary Fig. 2).

Climate variability is indeed a well-known driver of human mortality, which mainly accounts for the impact of temperature fluctuations both at seasonal (e.g., winter and summer mortality, influenza season^52^) and daily (e.g., cold spells, heat waves^50^) timescales. Our investigation of daily temperatures, mortality rates, and economic growth describes for the first time a new dimension of the procyclical oscillation of mortality trends, that is, the fact that the procyclical character of mortality varies throughout the calendar year, and therefore, it might also depend on the seasonal timing of the underlying causes of death (Fig. 4 and Supplementary Fig. 4). We found that mortality rates are procyclical for the whole range of temperatures, but that the strength of the association is somewhat larger for specific periods of the year. Up to our knowledge, this dependency has never been described to date. Although this is only a preliminary result deserving a more in-depth analysis, we generally found larger correlation values in autumn, winter, and spring than in summer (Fig. 4 and Supplementary Fig. 4). This result suggests that the strength of the association between macroeconomic expansion and increase in mortality in the cold part of the year may perhaps be determined by the seasonal character of the major causes of death, as for instance fatalities due to influenza and other respiratory diseases have a clear seasonality, rather than the direct effect of extreme temperature days and cold spells during the harshest weeks in winter. This effect could be in turn related with the interaction of these causes of death with some of the effects of recessions, such as changes in atmospheric pollution (i.e., better air quality during recessions would exacerbate to a lower extent pre-existing cardio-respiratory diseases), unemployment (larger rates would reduce the transmission of infectious diseases at the workplace), or bad habits (e.g., the reduction in the consumption of alcohol would improve the physiological response to these diseases).

We must note that we found generally lower correlation values in the lowest (below percentile 10) and highest (above percentile 90) temperatures, probably due to the lower frequency of climatic extremes. Their hazardous occurrence can generate non-representative, noisy values of the relationship between daily temperatures and mortality rates when relatively short subperiods are analyzed, as it can be for example observed as a result of the record-breaking summer 2003 heat wave in France (see the red curve in Supplementary Fig. 2h^53,54^). These rather rarer events are likely to modify the mortality trends at the extremes of the temperature distribution, and weaken the magnitude of the association between changes in the trends.

There is a growing body of literature quantifying the short-term impact (i.e., lags up to 1-4 weeks) of daily temperatures on daily mortality by means of DLNM techniques. These studies describe the relationship between temperature and the relative risk of death as asymmetric U-shaped curves, with generally monotonically increasing risks for temperatures above or below a temperature of minimum mortality at around percentiles 60–90^51^. Instead, in Supplementary Fig. 4 of the present study, we did not generally find this U-shaped pattern, but rather a more uniform dependency with temperatures. In our opinion, this mismatch is essentially explained by the very different nature of the two scientific questions (i.e., procyclical evolution of mortality and temperature-related mortality), which in turn have been traditionally addressed by means of very different methodological approaches (cf. refs. ^50,51^ with refs. ^4,11^).

The procyclical behavior of mortality shown in Fig. 4 and Supplementary Fig. 4 might be different in other areas of the world or in less developed countries. By design, our methodology captures the differences among European countries in social safety nets and air quality regulations, which might differently buffer the impacts of macroeconomic fluctuations. In Europe, differences might be seen between the Nordic countries (with higher development level, life expectancy, and acclimatization to cold weather) and the Euro-Mediterranean area, or between Western (with higher development level, life expectancy, and public resources in social services) and Eastern Europe. More interestingly, some authors are starting to show that the negative effect of rising temperatures on temperature-related mortality is being counterbalanced by an adaptive societal response, which is reducing the mortality risk associated to heat conditions in summer^50^. This long-term modification of the temperature-mortality relationship, with monotonically decreasing mortality risks over the last decades, is occurring in parallel with the recurrent effect of the macroeconomic cycle of expansion and recession. This issue will deserve further analyses, and although the burden of deaths directly attributable to cold and heat temperatures only represents up to about 10% of the overall mortality^51^, the procyclical evolution of mortality might have changed, or might now change, as a result of the current context of rising temperatures and the associated adaptive response of societies.

## Methods

### Mortality and population data

Daily counts of all-cause mortality and annual population estimates by sex and 5-year age groups (0–4, 5–9, …, 74–79, 80+) were collected between the years 2000 and 2010 for 140 regions in 15 European countries representing 400 million people, namely Austria (acronym AT, with data in nine regions), Belgium (BE, 11), Croatia (HR, 2), the Czech Republic (CZ, 8), Denmark (DK, 1), France (FR, 22), Germany (DE, 16), Italy (IT, 21), Luxembourg (LU, 1), Netherlands (NL, 1), Poland (PL, 16), Portugal (PT, 5), Slovenia (SI, 1), Spain (ES, 16), and the United Kingdom (UK, 10 regions in England and Wales only). Daily population numbers were estimated with a 5-step protocol based on the lexis diagram, which integrates daily age specific mortality counts with annual population numbers of consecutive age strata (full methodological details can be found in ref. ^55^) for age strata above 64 and with a linear interpolation between two consecutive years for age strata below 65.

### Standardization of mortality data

Daily standardized mortality rates were computed by using direct standardization by sex and 5-year age groups, with the reference population being the total number of residents present in all the 15 countries, on 31 December 2005. When crude mortality rates are standardized, they have a steeper declining slope, because the mortality-increasing effect of aging that flattens crude mortality is eliminated in the adjusted rate (Fig. 1, Supplementary Fig. 5 and Table 2). Only standardized mortality rates are used in the article. Mortality data by sex and age groups was not available in Croatia and the United Kingdom for years 2000 and 2001, and therefore these years were excluded from the analyses in these two particular cases. Apart from that, the methodology used to collect or estimate death and population data was strictly identical in all the countries, and therefore no methodological and/or data quality issue can explain differences across national or regional borders.

### GDP and climate data

Annual per capita estimates of real GDP by country (with reference price levels from year 2010, Table 1) and GDP at current market prices by country and region were obtained from Eurostat. The relationship between the two measures of GDP is essentially linear in each country, mainly due to the low levels of inflation in Europe during the study period (Supplementary Fig. 6). Given that data of real GDP is not available at the regional level, we used GDP at current market prices unless otherwise specified. Daily high-resolution gridded (0.25° × 0.25°) observations of daily mean 2-meter temperature were derived from E-OBS v14, and transformed into regional estimates^52,53^.

### Changes in GDP and mortality trends

The effect of the economic crisis on annual mortality trends is shown in Fig. 2, in which we averaged the relative year-to-year changes in annual GDP and mortality over the expansion and recession periods. Thus, we defined the trend of a variable *v* in a country or region *r* over the period between years *y*_1_ and *y*_2_ as

1 $${t_{y_1,y_2}^r(v) = \frac{1}{{y_2 - y_1 + 1}}\mathop {\sum}\limits_{y = y_1}^{y_2} {\frac{{v_y^r - v_{y - 1}^r}}{{(v_y^r + v_{y - 1}^r)/2}}} },$$

i.e., the relative year-to-year changes in variable *v* averaged over the years between *y*_1_ and *y*_2_. We then computed the post- minus pre-crisis change in the trends, i.e.,

2 $${{{\mathrm{\Delta }}t_{\mathrm{crisis}}^r(v) = t_{{\mathrm{post}} - {\mathrm{crisis}}}^r(v) - t_{{\mathrm{pre}} - {\mathrm{crisis}}}^r(v) = t_{2008,2010}^r(v) - t_{2001,2007}^r(v)}} ,$$

of annual GDP $\left( {{{\mathrm{\Delta }}t_{\mathrm{crisis}}^r{\mathrm{(GDP)}}}} \right)$ and mortality $\left( {{{\mathrm{\Delta }}t_{\mathrm{crisis}}^r(M)}} \right)$, which are shown in Fig. 2 separately for the ensemble of countries (in red) and regions (in cyan).

### Changes in mortality trends by temperature percentile

For the calculation of the relationship between daily temperatures and mortality rates, we chose the time lag in order to maximize the amount of explained variance, i.e., 1 week from October to May and unlagged from June to September. The temperature axis was then divided in equally spaced intervals, the set of days belonging to each interval grouped, and daily temperatures and mortality rates averaged. The resulting relationship between interval mean temperatures and mortality rates is shown for the ensemble of countries (*r*) and three subperiods (*y*_1_, *y*_2_) as solid curves in Supplementary Fig. 2, and used to transform temperatures into mortality estimations $\left( {{M_{y_1,y_2}^r = f_{y_1,y_2}^r(T)}} \right)$. We note that these mortality estimations correspond to the expected mortality for a day with a given temperature *T*, in a given location *r*, and within a given period (*y*_1_, *y*_2_). We clarify that this is not the expected mortality directly attributed to temperature, i.e. temperature-related mortality, but rather a method to compute changes in mortality trends expressed as a function of daily temperature.

Thus, for each country or region *r*, we computed percentiles *p* *=* 0, 1,…, 100 of the daily temperature time series of the overall period 2000–2010 $\left( {{T_p^r}} \right)$, and transformed them into expected mortality estimations by using the daily temperature/mortality relationship from selected subperiods (${M_{y_1,y_2}^{r,p} = f_{y_1,y_2}^r(T_p^r)}$, Supplementary Fig. 2). Differences between consecutive subperiods were used to infer pre- and post-crisis mortality trends, i.e.

3 $${\hat t_{{{\mathrm{pre}} -{\mathrm{crisis}}}}^{r,p}(M) = (M_{2004,2007}^{r,p} - M_{2000,2003}^{r,p}})/4$$

and

4 $${\left. {\hat t_{{\mathrm{post} -{\mathrm{crisis}}}}^{r,p}(M) = (M_{2008,2010}^{r,p} - M_{2004,2007}^{r,p})/3.5} \right),}$$

and the change between these trends to estimate the effect of the economic crisis by temperature percentile, i.e.,

5 $${{\mathrm{\Delta }}\hat t_{\mathrm{crisis}}^{r,p}(M) = \hat t_{{\mathrm{post}} - {\mathrm{crisis}}}^{r,p}(M) - \hat t_{{\mathrm{pre}} -{{\mathrm{crisis}}}}^{r,p}(M)}$$

(Figure 3 and Supplementary Fig. 3). We note that trends were divided by the number of years between consecutive subperiods, i.e., 4 for the pre-crisis and 3.5 for the post-crisis. The association between these changes in mortality trends $\left( {{{\mathrm{\Delta }}\hat t_{\mathrm{crisis}}^{r,p}(M)}} \right)$ and the change in annual GDP trend $\left( {{{\mathrm{\Delta }}t_{\mathrm{crisis}}^r{\mathrm{(GDP)}}}} \right.$, i.e., the one already shown in the *x*-axis of Fig. 2), are depicted in Fig. 4 and Supplementary Fig. 4 separately for the ensemble of countries (in red) and regions (in cyan) as Pearson correlations by temperature percentile *p*.

### Test of statistical significance

The distribution of daily mortality data within each equally spaced temperature interval in the temperature/mortality relationships (see the corresponding 95% confidence interval as dashed lines in Fig. 3 and Supplementary Figs 2 and 3) was used to generate 1000 Monte Carlo simulations and test the statistical significance of the Pearson correlations.

## Supplementary Information

Supplementary Information

## Data Availability

The climate data can be obtained from the European Climate Assessment and Dataset (ECA&D, www.ecad.eu), and the GDP data from Eurostat (ec.europa.eu/eurostat). The authors do not have permission to directly share the third party mortality and population data used. The code is available on request.
