# Supplementary material for: Effect of the Great Recession on regional mortality trends in Europe
Source: Nat Commun. 2019 Feb 8;10:679. doi: 10.1038/s41467-019-08539-w (PMC6368579; doi:10.1038/s41467-019-08539-w)
Supplement: Supplementary file 1 — Supplementary Information [file 41467_2019_8539_MOESM1_ESM.pdf]

Title:

Effect of the Great Recession on regional mortality trends in Europe

Authors:

Joan Ballester (1)
Jean-Marie Robine (2,3)
François R. Herrmann (4)
Xavier Rodó (1,5)

Affiliations:

(1) Climate and Health Program, Barcelona Institute for Global Health (ISGlobal), Barcelona, Catalonia, Spain
(2) Institut National de la Santé et de la Recherche Médicale (INSERM), Montpellier, France
(3) École Pratique des Hautes Études, Paris, France
(4) Division of Geriatrics, Department of Rehabilitation and Geriatrics, Thônex, Geneva University Hospitals and University of Geneva, Switzerland
(5) ICREA, Barcelona, Catalonia, Spain

Corresponding author:

Joan Ballester
Barcelona Institute for Global Health (ISGlobal)
Carrer Doctor Aiguader 88, Office 186, 08003 Barcelona, Catalonia, Spain
Tel.: (+34) 932147348
Email: joan.ballester@isglobal.org

Nature Communications − Supplementary Material

Supplementary Figure 1. **Recession minus expansion change in trends**.

The panel shows the difference (2006-2010 minus 2001-2005) in average relative year-to-year changes (%) of annual Gross Domestic Product (GDP) and age-standardized mortality by country (red squares) and region (cyan triangles). Correlations are depicted in the top-left corner (Pearson first, Spearman in brackets). Country acronyms are: Austria (AT), Belgium (BE), Croatia (HR), the Czech Republic (CZ), Denmark (DK), France (FR), Germany (DE), Italy (IT), Luxembourg (LU), Netherlands (NL), Poland (PL), Portugal (PT), Slovenia (SI), Spain (ES) and the United Kingdom (UK).

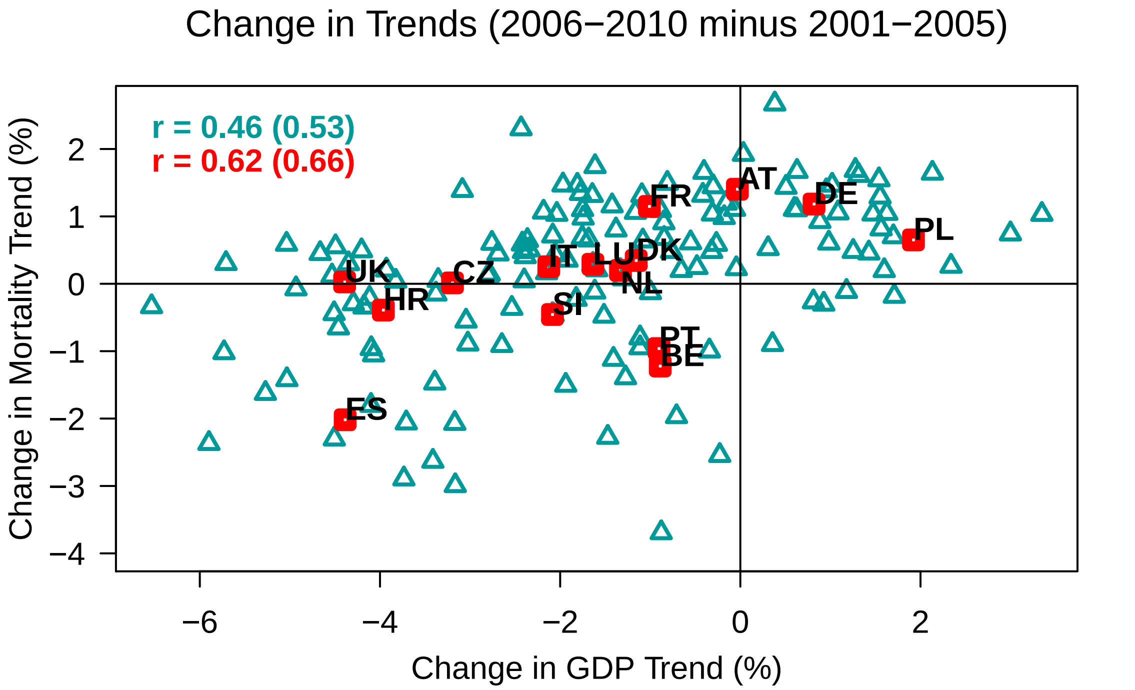

Change in Trends (2006−2010 minus 2001−2005)
r = 0.46 (0.53)
r = 0.62 (0.66)
Change in Mortality Trend (%)
Change in GDP Trend (%)
2
1
0
−1
−2
−3
−4
−6
−4
−2
0
2
AT
FR
DE
PL
IT
LU
DK
UK
CZ
NL
HR
SI
PT
BE
ES

Supplementary Figure 2. **Relationship between daily temperature and mortality rates**.

The panels show the relationship between daily temperatures (°C) and age-standardized mortality rates (daily deaths per million). Dots correspond to the relationship for subperiods 2000-2003 (red), 2004-2007 (green) and 2008-2010 (blue). Solid and dashed curves correspond to the average and 95% confidence interval of daily mortality rates within each equally spaced temperature interval, respectively.

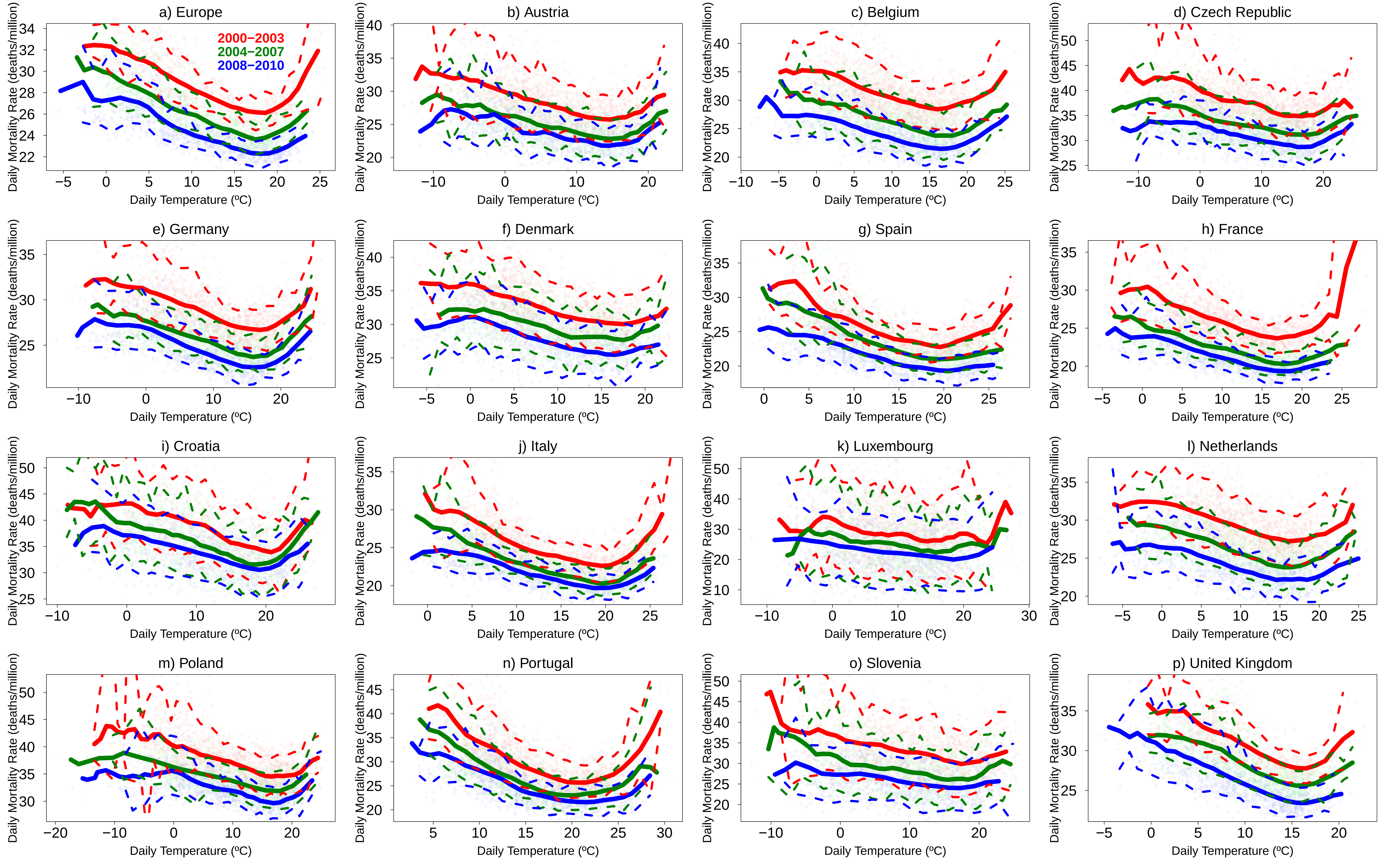

a) Europe
b) Austria
c) Belgium
d) Czech Republic
e) Germany
f) Denmark
g) Spain
h) France
i) Croatia
j) Italy
k) Luxembourg
l) Netherlands
m) Poland
n) Portugal
o) Slovenia
p) United Kingdom
2000–2003
2004–2007
2008–2010
Daily Mortality Rate (deaths/million)
Daily Temperature (ºC)

Supplementary Figure 3. **Change in mortality trends by temperature percentile**.

Panels show the change in the trend of age-standardized mortality by temperature percentile. For each location, percentiles of the daily temperature time series of the overall period 2000-2010 were computed and transformed into mortality estimations by using the daily temperature/mortality relationship from subperiods 2000-2003 (P1), 2004-2007 (P2) and 2008-2010 (P3). Differences in mortality between consecutive subperiods were used to infer expansion (P2 minus P1) and recession (P3 minus P2) trends (daily deaths per million per year). Solid lines correspond to the recession minus expansion difference in trends, and dashed curves to the 95% confidence interval.

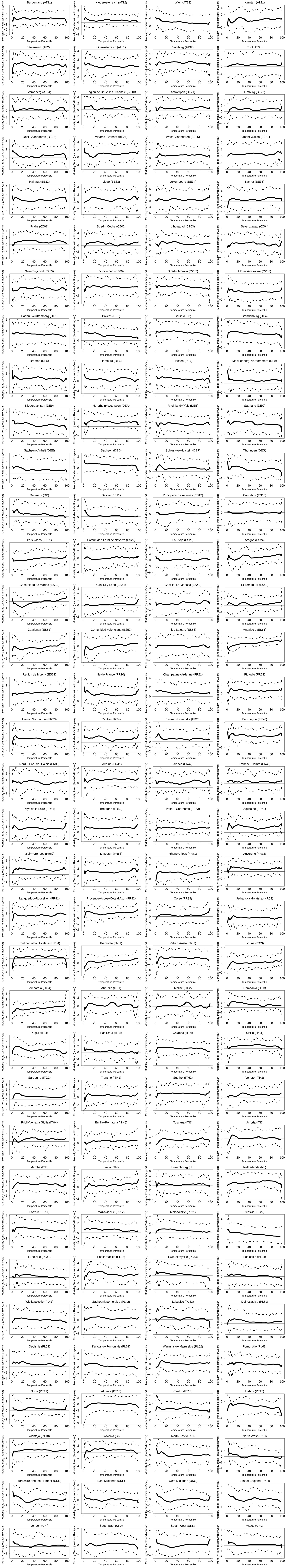
Burgenland (AT11)
Niederosterreich (AT12)
Wien (AT13)
Karnten (AT21)
Steiermark (AT22)
Oberosterreich (AT31)
Salzburg (AT32)
Tirol (AT33)
Vorarlberg (AT34)
Region de Bruxelles−Capitale (BE10)
Antwerpen (BE21)
Limburg (BE22)
Oost−Vlaanderen (BE23)
Vlaams−Brabant (BE24)
West−Vlaanderen (BE25)
Brabant Wallon (BE31)
Hainaut (BE32)
Liege (BE33)
Luxembourg (BE34)
Namur (BE35)
Praha (CZ01)
Stredni Cechy (CZ02)
Jihozapad (CZ03)
Severozapad (CZ04)
Severovychod (CZ05)
Jihovychod (CZ06)
Stredni Morava (CZ07)
Moravskoslezsko (CZ08)
Baden−Wurttemberg (DE1)
Bayern (DE2)
Berlin (DE3)
Brandenburg (DE4)
Bremen (DE5)
Hamburg (DE6)
Hessen (DE7)
Mecklenburg−Vorpommern (DE8)
Niedersachsen (DE9)
Nordrhein−Westfalen (DEA)
Rheinland−Pfalz (DEB)
Saarland (DEC)
Sachsen−Anhalt (DEE)
Sachsen (DED)
Schleswig−Holstein (DEF)
Thuringen (DEG)
Denmark (DK)
Galicia (ES11)
Principado de Asturias (ES12)
Cantabria (ES13)
Pais Vasco (ES21)
Comunidad Foral de Navarra (ES22)
La Rioja (ES23)
Aragon (ES24)
Comunidad de Madrid (ES30)
Castilla y Leon (ES41)
Castilla−La Mancha (ES42)
Extremadura (ES43)
Cataluna (ES51)
Comunidad Valenciana (ES52)
Illes Balears (ES53)
Andalucia (ES61)
Region de Murcia (ES62)
Ile de France (FR10)
Champagne−Ardenne (FR21)
Picardie (FR22)
Haute−Normandie (FR23)
Centre (FR24)
Basse−Normandie (FR25)
Bourgogne (FR26)
Nord − Pas−de−Calais (FR30)
Lorraine (FR41)
Alsace (FR42)
Franche−Comte (FR43)
Pays de la Loire (FR51)
Bretagne (FR52)
Poitou−Charentes (FR53)
Aquitaine (FR61)
Midi−Pyrenees (FR62)
Limousin (FR63)
Rhone−Alpes (FR71)
Auvergne (FR72)
Languedoc−Roussillon (FR81)
Provence−Alpes−Cote d'Azur (FR82)
Corse (FR83)
Jadranska Hrvatska (HR03)
Kontinentalna Hrvatska (HR04)
Piemonte (ITC1)
Valle d'Aosta (ITC2)
Liguria (ITC3)
Lombardia (ITC4)
Abruzzo (ITF1)
Molise (ITF2)
Campania (ITF3)
Puglia (ITF4)
Basilicata (ITF5)
Calabria (ITF6)
Sicilia (ITG1)
Sardegna (ITG2)
Trentino (ITH1)
Sudtirol (ITH2)
Veneto (ITH3)
Friuli−Venezia Giulia (ITH4)
Emilia−Romagna (ITH5)
Toscana (ITI1)
Umbria (ITI2)
Marche (ITI3)
Lazio (ITI4)
Luxembourg (LU)
Netherlands (NL)
Lodzkie (PL11)
Mazowieckie (PL12)
Malopolskie (PL21)
Slaskie (PL22)
Lubelskie (PL31)
Podkarpackie (PL32)
Swietokrzyskie (PL33)
Podlaskie (PL34)
Wielkopolskie (PL41)
Zachodniopomorskie (PL42)
Lubuskie (PL43)
Dolnoslaskie (PL51)
Opolskie (PL52)
Kujawsko−Pomorskie (PL61)
Warminsko−Mazurskie (PL62)
Pomorskie (PL63)
Norte (PT11)
Algarve (PT15)
Centro (PT16)
Lisboa (PT17)
Alentejo (PT18)
Slovenia (SI)
North East (UKC)
North West (UKD)
Yorkshire and the Humber (UKE)
East Midlands (UKF)
West Midlands (UKG)
East of England (UKH)
London (UKI)
South East (UKJ)
South West (UKK)
Wales (UKL)
Temperature Percentile
Mortality Trend (deaths/million/year)

Supplementary Figure 4. **Change in trends by temperature percentile**.

Curves correspond to the Pearson correlations (a) and p-values (b) between changes in the trends of Gross Domestic Product (GDP) and age-standardized mortality by temperature percentile and for the ensemble of countries (red) and regions (cyan). As a visual reference, horizontal dashed lines correspond to the overall Pearson correlations.

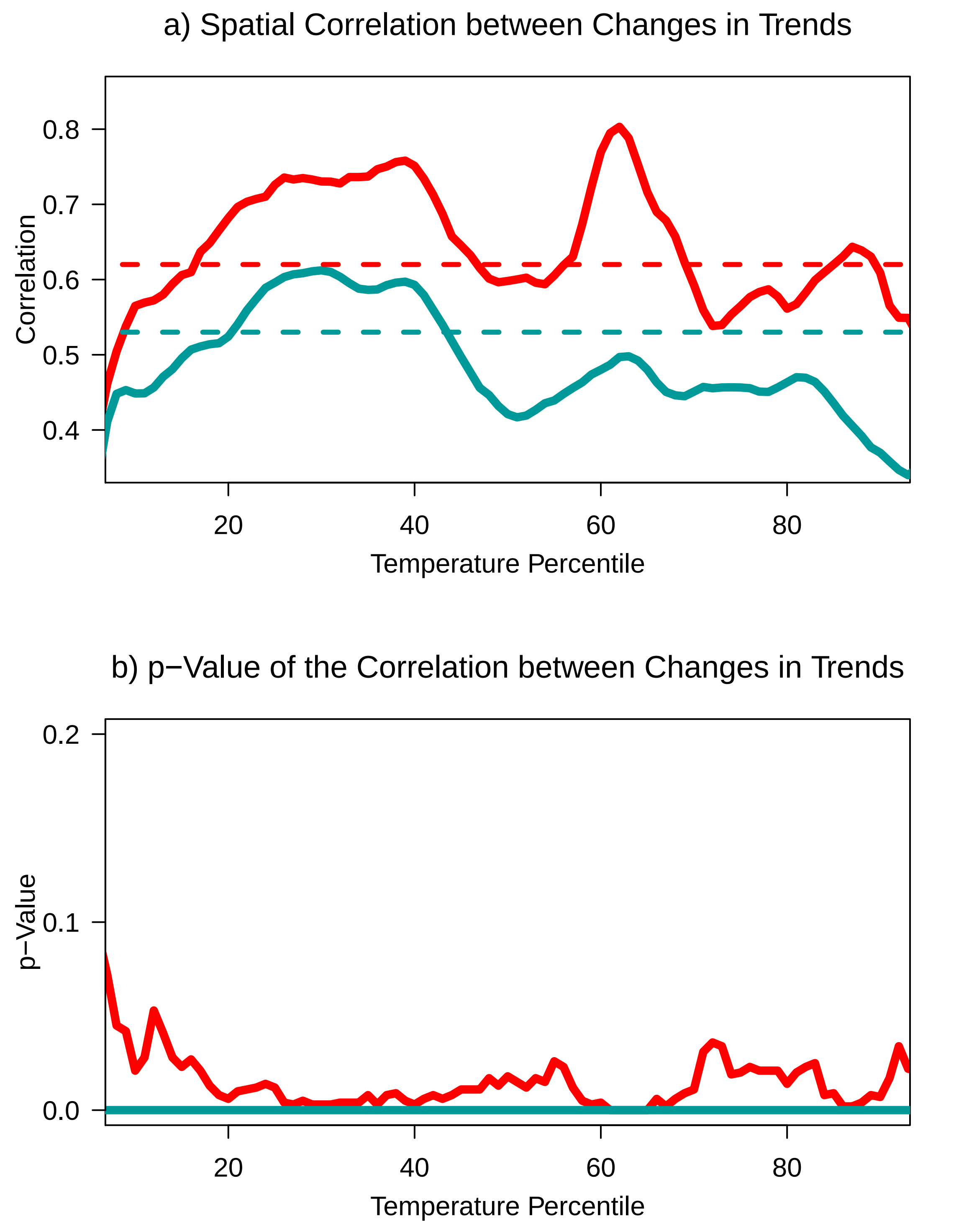
a) Spatial Correlation between Changes in Trends
Correlation
0.8
0.7
0.6
0.5
0.4
20
40
60
80
Temperature Percentile
b) p−Value of the Correlation between Changes in Trends
p−Value
0.2
0.1
0.0
20
40
60
80
Temperature Percentile

Supplementary Figure 5. **Relationship between crude and age-standardized mortality**.

Circles correspond to relative year-to-year changes (%) in annual mortality by country. The linear least squares fitting (dashed line) and Pearson correlation are depicted in the top-left corner. The color legend is provided in Figure 1.

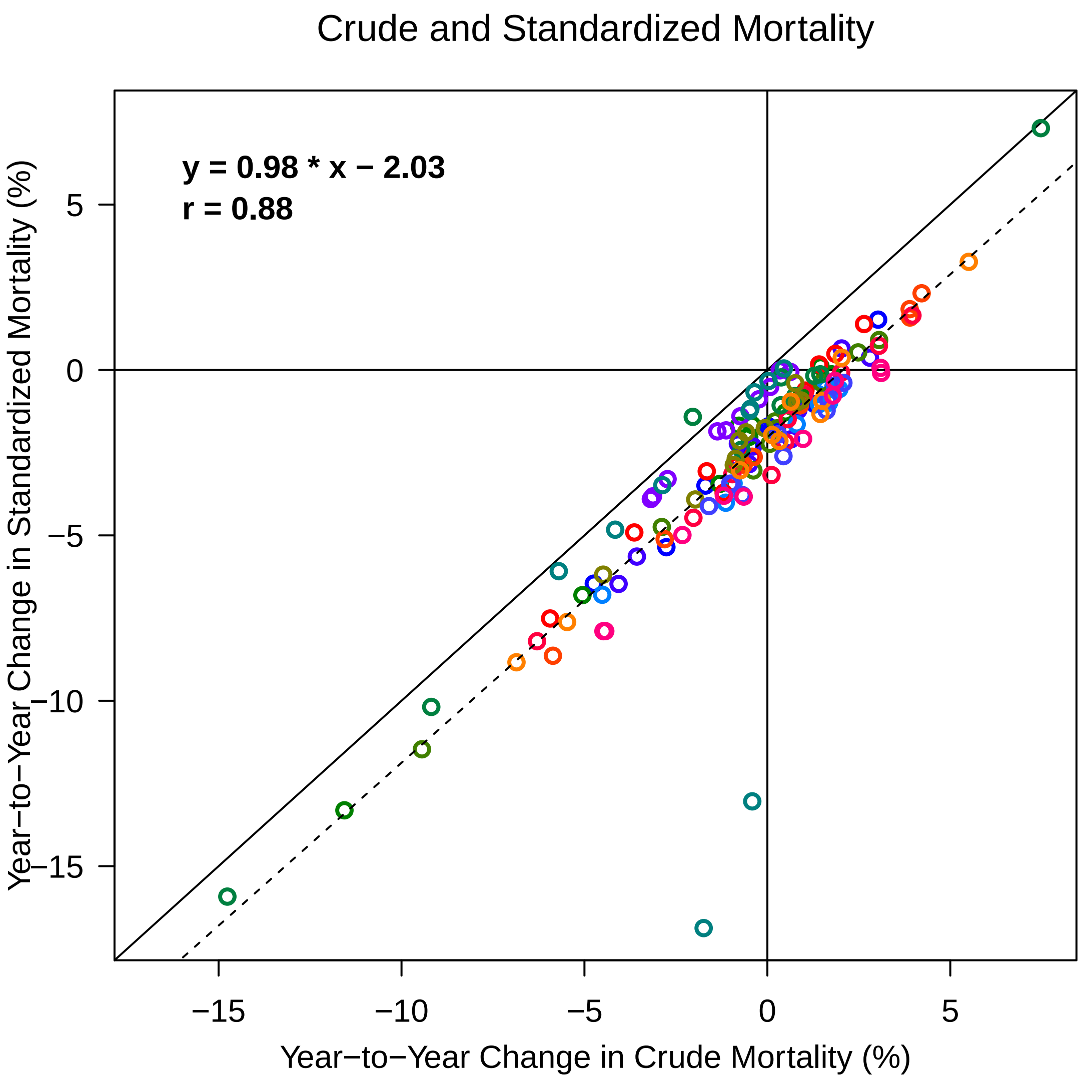
Crude and Standardized Mortality
y = 0.98 * x − 2.03
r = 0.88
Year−to−Year Change in Standardized Mortality (%)
Year−to−Year Change in Crude Mortality (%)
5
0
−5
−10
−15
−15
−10
−5
0
5

Supplementary Figure 6. **Comparison between Gross Domestic Product estimates**.

The panel shows the relationship between Gross Domestic Product (GDP) at current market prices and real GDP. Circles correspond to annual estimates of GDP (thousand euros) by country. Pearson correlations are depicted next to the corresponding country acronym: Austria (AT), Belgium (BE), Croatia (HR), the Czech Republic (CZ), Denmark (DK), France (FR), Germany (DE), Italy (IT), Luxembourg (LU), Netherlands (NL), Poland (PL), Portugal (PT), Slovenia (SI), Spain (ES) and the United Kingdom (UK).

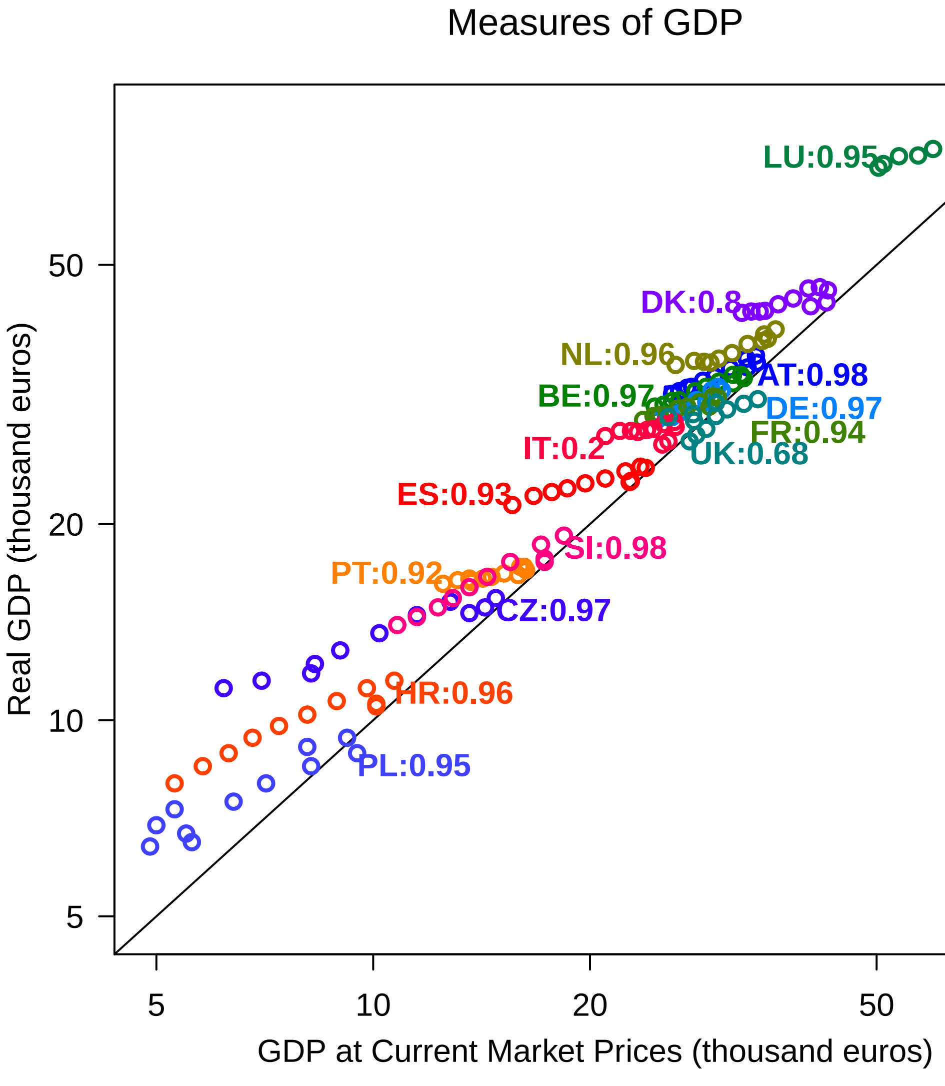

Measures of GDP
LU:0.95
DK:0.8
NL:0.96
AT:0.98
BE:0.97
DE:0.97
FR:0.94
IT:0.2
UK:0.68
ES:0.93
SI:0.98
PT:0.92
CZ:0.97
HR:0.96
PL:0.95
Real GDP (thousand euros)
50
20
10
5
GDP at Current Market Prices (thousand euros)
5
10
20
50
